# O Melhor do Ano 2020 nos Arquivos Brasileiros de Cardiologia e na Revista Portuguesa de Cardiologia

**DOI:** 10.36660/abc.20210372

**Published:** 2021-06-08

**Authors:** Ricardo Fontes-Carvalho, Gláucia Maria Moraes de Oliveira, Nuno Cardim, Carlos Eduardo Rochitte

**Affiliations:** 1 Departamento de Cardiologia Centro Hospitalar de Vila Nova de Gaia/Espinho Vila Nova de Gaia Portugal Departamento de Cardiologia – Centro Hospitalar de Vila Nova de Gaia/Espinho , Vila Nova de Gaia - Portugal; 2 Departamento de Cirurgia e Fisiologia Faculdade de Medicina Universidade do Porto Porto Portugal Departamento de Cirurgia e Fisiologia – Faculdade de Medicina – Universidade do Porto , Porto - Portugal; 3 Faculdade de Medicina Universidade Federal do Rio de Janeiro Rio de JaneiroRJ Brasil Faculdade de Medicina – Universidade Federal do Rio de Janeiro , Rio de Janeiro , RJ - Brasil; 4 Instituto do Coração Edson Saad Universidade Federal do Rio de Janeiro Rio de JaneiroRJ Brasil Instituto do Coração Edson Saad – Universidade Federal do Rio de Janeiro , Rio de Janeiro , RJ - Brasil; 5 Instituto do Coração Faculdade de Medicina Universidade de São Paulo São PauloSP Brasil Instituto do Coração (InCor) – Faculdade de Medicina da Universidade de São Paulo , São Paulo , SP - Brasil; 6 Hospital do Coração São PauloSP Brasil Hospital do Coração (HCOR), São Paulo , SP - Brasil; 7 Hospital da Luz-Lisboa Faculdade de Ciências Médicas Universidade Nova de Lisboa Lisboa Portugal Hospital da Luz-Lisboa - Faculdade de Ciências Médicas da Universidade Nova de Lisboa , Lisboa - Portugal

**Keywords:** Doenças Cardiovasculares, Cooperação Técnica/tendências, Intercâmbio de Pesquisadores/tendências, Disseminação da Informação, Fator de Impacto, Publicações Periódicas, Portugal, Brasil

## Introdução

Nos últimos anos a *Revista Portuguesa de Cardiologia* (RPC) e os *Arquivos Brasileiros de Cardiologia* (ABC Cardiol) têm cooperado na elaboração da lista dos melhores artigos publicados nas duas revistas, ^1 , 2^ dando assim destaque a alguns dos melhores trabalhos científicos realizados por autores de língua portuguesa.

Dado o sucesso dessa iniciativa, os corpos editoriais das duas revistas decidiram voltar a cooperar para elaborar a lista dos 10 melhores artigos publicados em 2020 em cada um desses periódicos. O ano de 2020 foi marcado pelo enorme impacto da pandemia de COVID-19 nos dois países, com uma elevada pressão sobre as instituições e profissionais de saúde. Apesar desses desafios, a qualidade científica das publicações nas duas revistas continuou muito elevada, com a divulgação de excelentes artigos originais.

A tarefa de seleção das melhores publicações é sempre complexa e, por vezes, pode ser injusta e imperfeita, sendo um processo independente das citações obtidas pelos respectivos artigos. Nesta seleção foram incluídos apenas artigos originais, não tendo sido considerados os de revisão. As tabelas 1 e 2 apresentam resumidamente os mais relevantes de 2020. Em seguida, fazemos um resumo dos principais resultados de cada um desses estudos e uma integração da relevância científica desses trabalhos.

**Tabela 1:** – Lista com a seleção dos dez melhores artigos publicados na *Revista Portuguesa de Cardiologia* em 2020

Autores	Título do artigo
D Abreu et al. ^3^	Impact of public health initiatives on acute coronary syndrome fatality rates in Portugal
H Dores et al. ^6^	Coronary atherosclerotic burden in veteran male recreational athletes with low to intermediate cardiovascular risk
D Roque et al. ^15^	Understanding a woman's heart: Lessons from 14 177 women with acute coronary syndrome
JP Moura Guedes et al. ^19^	P2Y12 inhibitor loading dose before catheterization in ST-segment elevation myocardial infarction: Is this the best strategy?
J Santos-Faria et al. ^24^	MicroRNAs and ventricular remodeling in aortic stenosis
C Guerreiro et al. ^26^	Short and long-term clinical impact of transcatheter aortic valve implantation in Portugal according to different access routes: Data from the Portuguese National Registry of TAVI
R Fontes-Carvalho et al. ^27^	Present and future economic impact of transcatheter aortic valve replacement on the Portuguese national healthcare system
M Gouveia et al. ^28^	Current costs of heart failure in Portugal and expected increases due to population aging
E Ozenc et al. ^29^	Impact of right ventricular stroke work index on predicting hospital readmission and functional status of patients with advanced heart failure
M Nobre Menezes et al. ^31^	Transradial left ventricular endomyocardial biopsy feasibility, safety and clinical usefulness: Initial experience of a tertiary university center

**Tabela 2:** – Lista com a seleção dos dez melhores artigos publicados nos *Arquivos Brasileiros de Cardiologia em 2020*

Autores	Título do artigo
Soriano L ^38^	1. Volume Vascular Pulmonar Estimado por Software Automatizado é um Preditor de Mortalidade após Embolia Pulmonar Aguda Pulmonary Vascular Volume Estimated by Automated Software is a Mortality Predictor after Acute Pulmonary Embolism http://abccardiol.org/wp-content/uploads/articles_xml/1678-4170-abc-115-05-0809/1678-4170-abc-115-05-0809.x64000.pdf http://abccardiol.org/wp-content/uploads/articles_xml/1678-4170-abc-115-05-0809/1678-4170-abc-115-05-0809-en.x64000.pdf
Oliveira-Junior SA ^11^	2. Bloqueio de Receptores AT _1_ Melhora o Desempenho Funcional Miocárdico na Obesidade AT _1_ Receptor Blockade Improves Myocardial Functional Performance in Obesity http://abccardiol.org/wp-content/uploads/articles_xml/0066-782X-abc-115-01-0017/0066-782X-abc-115-01-0017.x64000.pdf http://abccardiol.org/wp-content/uploads/articles_xml/0066-782X-abc-115-01-0017/0066-782X-abc-115-01-0017-en.x64000.pdf
Salim TR ^35^	3. Desigualdades nas Taxas de Mortalidade por Malformações do Sistema Circulatório em Crianças Menores de 20 Anos de Idade entre Macrorregiões Brasileiras Inequalities in Mortality Rates from Malformations of Circulatory System Between Brazilian Macroregions in Individuals Younger Than 20 Years http://abccardiol.org/wp-content/uploads/articles_xml/0066-782X-abc-115-06-1164/0066-782X-abc-115-06-1164.x64000.pdf http://abccardiol.org/wp-content/uploads/articles_xml/0066-782X-abc-115-06-1164/0066-782X-abc-115-06-1164-en.x64000.pdf
Mejia OAV ^36^	4. Análise de >100.000 Cirurgias Cardiovasculares Realizadas no Instituto do Coração e a Nova Era com Foco nos Resultados Analysis of >100,000 Cardiovascular Surgeries Performed at the Heart Institute and a New Era of Outcomes http://abccardiol.org/wp-content/uploads/articles_xml/0066-782X-abc-S0066-782X2020000400603/0066-782X-abc-S0066-782X2020000400603-pt.x64000.pdf http://abccardiol.org/wp-content/uploads/articles_xml/0066-782X-abc-S0066-782X2020000400603/0066-782X-abc-S0066-782X2020000400603.x64000.pdf
Alves L ^22^	5. Hospitalização por Infarto Agudo do Miocárdio: Um Registro de Base Populacional Hospitalization for Acute Myocardial Infarction: A Population-Based Registry http://abccardiol.org/wp-content/uploads/articles_xml/1678-4170-abc-115-05-0916/1678-4170-abc-115-05-0916.x64000.pdf http://abccardiol.org/wp-content/uploads/articles_xml/1678-4170-abc-115-05-0916/1678-4170-abc-115-05-0916-en.x64000.pdf
Campos FA ^34^	6. Estudo Comparativo da Doença Coronariana Microvascular Causada por Doença de Chagas com Outras Etiologias Chagas Cardiomyopathy as the Etiology of Suspected Coronary Microvascular Disease. A Comparison Study with Suspected Coronary Microvascular Disease of Other Etiologies http://abccardiol.org/wp-content/uploads/articles_xml/0066-782X-abc-115-06-1094/0066-782X-abc-115-06-1094.x64000.pdf http://abccardiol.org/wp-content/uploads/articles_xml/0066-782X-abc-115-06-1094/0066-782X-abc-115-06-1094-en.x64000.pdf
Stefan GP ^32^	7. Quantificação de Dano em DNA em Diferentes Tecidos em Ratos com Insuficiência Cardíaca Quantification of DNA Damage in Different Tissues in Rats with Heart Failure http://abccardiol.org/wp-content/uploads/articles_xml/0066-782X-abc-S0066-782X2020000200234/0066-782X-abc-S0066-782X2020000200234-pt.x64000.pdf http://abccardiol.org/wp-content/uploads/articles_xml/0066-782X-abc-S0066-782X2020000200234/0066-782X-abc-S0066-782X2020000200234.x64000.pdf
Basilio PG ^10^	8. Dieta Intermitente Atenua a Remodelação Cardíaca Causada pelo Exercício Físico Intermittent Fasting Attenuates Exercise Training-Induced Cardiac Remodeling http://abccardiol.org/wp-content/uploads/articles_xml/0066-782X-abc-115-02-0184/0066-782X-abc-115-02-0184.x64000.pdf http://abccardiol.org/wp-content/uploads/articles_xml/0066-782X-abc-115-02-0184/0066-782X-abc-115-02-0184-en.x64000.pdf
Santos JL ^12^	9. Os Percentis e Pontos de Corte da Circunferência Abdominal para Obesidade em uma Ampla Amostra de Estudantes de 6 a 10 Anos de Idade do Estado de São Paulo, Brasil Waist Circumference Percentiles and Cut-Off Values for Obesity in a Large Sample of Students from 6 To 10 Years Old Of The São Paulo State, Brazil http://abccardiol.org/wp-content/uploads/articles_xml/0066-782X-abc-114-03-0530/0066-782X-abc-114-03-0530.x64000.pdf http://abccardiol.org/wp-content/uploads/articles_xml/0066-782X-abc-114-03-0530/0066-782X-abc-114-03-0530-en.x64000.pdf
Ferreira LCM ^21^	10. Mortalidade por Infarto Agudo do Miocárdio no Brasil de 1996 a 2016: 21 Anos de Contrastes nas Regiões Brasileiras Mortality Due to Acute Myocardial Infarction in Brazil from 1996 to 2016: 21 Years of Disparities in Brazilian Regions http://abccardiol.org/wp-content/uploads/articles_xml/1678-4170-abc-115-05-0849/1678-4170-abc-115-05-0849.x64000.pdf http://abccardiol.org/wp-content/uploads/articles_xml/1678-4170-abc-115-05-0849/1678-4170-abc-115-05-0849-en.x64000.pdf

## Prevenção e risco cardiovascular

A doença cardiovascular (DCV) é a principal causa de morte em Portugal, no Brasil e nos países desenvolvidos, sendo fundamental a implementação de medidas de saúde pública dirigidas à população em geral que possam reduzir o impacto da DCV na sociedade. Durante a década 2000-2010, Portugal implementou um conjunto de políticas de saúde pública para tentar reduzir a mortalidade por DCV. Foram exemplos dessas medidas a lei do tabaco de 2008, a lei de redução do sal de 2010 e a implementação da via verde coronária (VVC) em 2007. Num dos estudos publicados em 2020 na RPC, Abreu et al., ^3^ pretenderam avaliar o impacto dessas três políticas de saúde na redução das taxas de letalidade por síndrome coronariana aguda (SCA), analisando os dados epidemiológicos da DCV entre 2000 e 2016. Os resultados desse estudo sugerem que a lei do tabaco de 2008 e a implementação da VVC levaram a uma diminuição imediata da taxa de letalidade por SCA, efeito que não foi observado com a lei do sal de 2010. Sabemos que a redução do consumo de sal tem sobretudo impacto na redução do risco de acidente vascular cerebral, o que pode explicar os resultados desse estudo. Além disso, esses dados são consistentes com aqueles observados no Registro Nacional de SCA da Sociedade Portuguesa de Cardiologia, que mostram um aumento consistente das taxas de revascularização após a implementação da VVC. ^4^ Curiosamente, também no ano de 2020, um outro artigo publicado na RPC mostrou que a proibição abrangente do tabagismo na comunidade de Valência (Espanha) associou-se a uma forte redução das taxas ajustadas de internamento por infarto do miocárdio. ^5^ Esse tipo de estudo e análise são fundamentais para que se possam implementar novas medidas de saúde pública capazes de reduzir o impacto da DCV na sociedade.

Ainda na área do risco cardiovascular (CV), Dores et al., ^6^ publicaram um interessante estudo na RPC em que pretenderam avaliar a carga de doença coronária aterosclerótica em atletas veteranos do gênero masculino assintomáticos com risco CV baixo-intermédio. Para isso, realizaram tomografia computorizada (TC) cardíaca com determinação do escore de cálcio e angiotomografia computadorizada (Angio-TC) coronária em 105 atletas. Apesar de essa população parecer a princípio “saudável”, esse estudo mostrou uma carga aterosclerótica coronária elevada em 25,7% dos indivíduos e lesões coronárias obstrutivas em 5,7%. Nesse estudo não se observou uma relação entre a extensão e a gravidade das placas coronárias e a quantidade ou o tipo de exercício praticado. Esses dados são importantes para ajudar a compreender as melhores estratégias de rastreio em atletas veteranos ^7^ e levantam a possibilidade de a TC coronária poder ser utilizada na avaliação de rotina desses indivíduos, conforme discutido no editorial de Pelliccia sobre esse artigo. ^8^

As taxas de mortalidade padronizadas por idade para DCV atribuíveis a fatores de risco em 2018, no Brasil, para mulheres e homens, classificam os riscos dietéticos no segundo lugar do *ranking* , atrás apenas da hipertensão arterial. ^9^ Basilio et al., ^10^ estudaram a influência da combinação da dieta intermitente (com restrição calórica) e exercício físico sobre a capacidade funcional, o metabolismo glicêmico e a remodelação cardíaca em ratos Wistar machos, durante 12 semanas. Sua hipótese era de que o exercício físico ampliaria o desempenho físico e atenuaria a remodelação miocárdica decorrente da restrição calórica intermitente. Os autores observaram que o exercício físico aumentou a capacidade funcional e acarretou fibrose cardíaca. Concluíram que a dieta intermitente se associou com melhor tolerância glicêmica e atenuou o processo de remodelação cardíaca decorrente do exercício físico, porém não interferiu na capacidade funcional.

O aumento do índice de massa corporal (IMC), representando as alterações observadas na obesidade, é o terceiro fator de risco para as mulheres e o quarto para os homens, no *ranking* anteriormente mencionado. ^9^ Oliveira-Júnior et al., ^11^ levantaram a hipótese de que a obesidade se associaria com alterações no desempenho funcional miocárdico sustentadas em diferentes condições de estimulação e que seriam reduzidas com o antagonismo de receptores AT1. Estudaram ratos Wistar que receberam dieta controle ou hiperlipídica e foram divididos de acordo com a presença de obesidade. Tanto o grupo de ratos obesos quanto o grupo controle receberam losartan (30 mg/kg/dia) na água durante quatro semanas. Os autores concluíram que a obesidade induzida por dieta promoveu a remodelação cardíaca, sustentada por hipertrofia ventricular e disfunção miocárdica, confirmando a hipótese inicial de que a estimulação de receptores AT1 está associada com prejuízos na função miocárdica nos ratos obesos. Losartan melhorou a função miocárdica de ratos com obesidade induzida por dieta.

É importante notar que a obesidade vem aumentando não só na população adulta, como em crianças e adolescentes ao redor do mundo. A medição da circunferência abdominal, que é um parâmetro de fácil obtenção, tem alta sensibilidade na predição de níveis de adiposidade visceral em crianças. Santos et al., ^12^ realizaram um estudo multicêntrico, prospectivo, transversal com 22.000 crianças (11.199 meninos) de 6 a 10 anos, matriculadas no ensino fundamental de escolas públicas e particulares de 13 cidades do estado de São Paulo. Aferiram a estatura, o peso e a circunferência abdominal. A prevalência de obesidade variou de 17% (6 anos de idade) a 21,6% (9 anos de idade), entre os meninos, e de 14,1% (7 anos de idade) a 17,3% (9 anos de idade), entre as meninas. Esse estudo corrobora os achados sobre obesidade infantil observados no ERICA ^13^ e chama atenção para a importância de intervenção precoce nos riscos dietéticos e na obesidade para evitar mortes CV na idade adulta.

## Doença coronária e síndrome coronariana aguda

Apesar de a mortalidade por doença coronária ter diminuído nos últimos anos, vários estudos relataram a persistência de grandes desigualdades de diagnóstico e tratamento da doença coronária de acordo com gênero, ^14^ sugerindo que as mulheres são frequentemente submetidas a pior tratamento. Num estudo publicado na RPC por Roque et al., ^15^ incluindo 49.113 pacientes (34.936 homens e 14.177 mulheres), os autores avaliaram as diferenças entre gêneros no tratamento da SCA utilizando os dados do Registro Nacional de SCA da Sociedade Portuguesa de Cardiologia. Observou-se que, em comparação com os homens, as mulheres são mais frequentemente admitidas por SCA sem supradesnivelamento do segmento ST e apresentam mais frequentemente sintomas atípicos. Talvez por isso, verificou-se que as mulheres têm tempos mais longos desde o início dos sintomas até receberem terapêutica de reperfusão. O risco de mortalidade intra-hospitalar foi significativamente maior no sexo feminino (OR 1,94; IC 95%: 1,78-2,12) assim como o risco de hemorragia maior, de insuficiência cardíaca (IC), de fibrilação atrial, de complicações mecânicas ou de choque cardiogênico. Foi ainda preocupante observar que as mulheres receberam menos frequentemente as terapias de prevenção secundária recomendadas, tanto durante a hospitalização como no momento da alta hospitalar. Em resumo, conforme discutido no editorial de Thomas Lüscher que acompanha esse artigo, ^16^ esses dados mostram a importância de se dar relevância ao tema da desigualdade entre gêneros no tratamento da DCV e focam a necessidade de serem implementadas medidas específicas que possam diminuir a disparidade de tratamento entre gêneros.

Em outro estudo publicado na RPC em 2020, também baseado nos dados do Registro Nacional de SCA da Sociedade Portuguesa de Cardiologia, os autores abordaram um dos grandes temas de discussão científica atual, ou seja o melhor *timing* para a administração do segundo antiplaquetário (inibidor da P2Y12) em pacientes com SCA com supradesnivelamento do segmento ST: administração pré-tratamento ou apenas no momento da angioplastia. ^17 , 18^ Para essa análise foram incluídos 4.123 pacientes com SCA, 66% dos quais foram medicados com o inibidor da P2Y12 antes da angioplastia. ^19^ Na análise multivariada, observou-se que os pacientes que receberam o inibidor da P2Y12 antes da angioplastia apresentaram aumento significativo do desfecho hemorrágico combinado (hemorragia maior, transfusão/queda de hemoglobina > 2g/dl), queda de hemoglobina > 2g/dl, e reinfarto, e nenhum benefício em termos de redução de eventos adversos CV maiores (MACE) ou morte intra-hospitalar. Esses dados são semelhantes aos observados em outros registros ^20^ e por isso contribuem para esta importante discussão.

O infarto agudo do miocárdio também é a principal causa de morte no Brasil, onde se observam disparidades regionais e por sexo nas tendências temporais das taxas de mortalidade nos anos mais recentes. Ferreira et al., ^21^ realizaram estudo de séries temporais de 21 anos, de 1996 a 2016, empregando dados do Sistema de Informações sobre Mortalidade (SIM) com correções de óbitos por causas mal definidas, códigos-lixo e sub-registro. Os autores observaram que a mortalidade diminuiu mais acentuadamente no sexo feminino (–2,2%; IC 95%: –2,5; –1,9) do que no masculino (–1,7%; IC 95%: –1,9; –1,4) e mais nas capitais (–3,8%; IC 95%: –4,3; –3,3) do que no interior (–1,5%; IC 95%: –1,8; –1,3). Verificaram ainda desigualdades regionais com aumento para homens residentes no interior do Norte (3,3; IC 95%: 1,3; 5,4) e Nordeste (1,3%; IC 95%: 1,0; 1,6). Concluíram que as correções dos números de óbitos são essenciais para estimativas mais fidedignas sobre as tendências das mortalidades por infarto do miocárdio no Brasil.

O infarto do miocárdio com supradesnivelamento do segmento ST (STEMI) apresenta a maior mortalidade proporcional entre as cardiopatias isquêmicas. Existem poucos estudos no Brasil de base populacional sobre as hospitalizações por essa causa. Alves L & Polanczyk CA ^22^ realizaram um estudo de coorte prospectiva de base populacional com registro consecutivo das hospitalizações por STEMI em uma cidade do Sul do Brasil, entre 2011 e 2014. Reportaram incidência anual de 108 casos/100.000 habitantes com taxa de reperfusão de 80,9%, mortalidade hospitalar de 8,9% e taxa de eventos CV de 6,1%. Concluíram que as hospitalizações foram em maior número do que nos países desenvolvidos, ainda que a abordagem terapêutica e a mortalidade hospitalar tenham sido semelhantes.

## Doenças valvares

A estenose valvar aórtica é atualmente a doença valvar mais frequente no mundo ocidental e a sua prevalência continuará a aumentar de forma exponencial devido ao envelhecimento da população. ^23^ A estenose aórtica causa alterações da estrutura e da função do ventrículo esquerdo, existindo vários mecanismos envolvidos na sua fisiopatologia. Num estudo publicado em 2020 na RPC por Santos-Faria et al., ^24^ os autores avaliaram o papel da modulação pós-transcricional por microARN no aparecimento de hipertrofia e fibrose do miocárdio. Analisando biópsias miocárdicas de 11 pacientes submetidos a cirurgia de substituição valvular aórtica, os autores observaram que o microARN-101-3p e o microARN-4268 poderão ter um novo papel na resposta hipertrófica em pacientes com estenose aórtica e que poderão ser usados como preditores de remodelagem reversa pós-cirurgia. Além disso, o papel desses microARN na regulação do sistema renina-angiotensina-aldosterona pode ajudar a descobrir novos alvos terapêuticos para a regressão da hipertrofia. ^25^

A implantação da válvula aórtica por via percutânea (TAVI) mudou o paradigma de tratamento da estenose aórtica grave. Em 2020, a RPC publicou um artigo que analisou os resultados a curto e longo prazo da TAVI em Portugal utilizando os dados do Registro Nacional de Cardiologia de Intervenção de Válvulas Aórticas Percutâneas (RNCI-VaP), numa análise de 2346 procedimentos. ^26^ No geral, TAVI associou-se a elevada eficácia e segurança, com uma taxa de mortalidade aos 30 dias de 4,8%. Os preditores de mortalidade aos 30 dias foram a doença arterial periférica, a presença de angioplastia prévia, de disfunção ventricular esquerda e a classe funcional NYHA III-IV. Os preditores de mortalidade em um ano foram a classe funcional NYHA III-IV, o acesso não transfemoral e a hemorragia com risco de vida. Os autores fizeram ainda uma análise interessante conforme o tipo de acesso efetuado (transfemoral ou outra via de acesso), mostrando que a abordagem transapical estava associada a maior mortalidade e maior risco de complicações, relacionada com um perfil de pacientes mais grave e com mais comorbidades.

Apesar dos seus benefícios, a penetração da TAVI em Portugal ainda é reduzida, com taxas bastante inferiores às da média da União Europeia. Além disso, a TAVI é uma terapêutica associada a custos elevados, sendo importante assegurar ao mesmo tempo a sustentabilidade do serviço nacional de saúde (SNS). Em outro artigo sobre o mesmo tema publicado na RPC em 2020, os autores pretenderam analisar o impacto econômico, atual e futuro, da TAVI em Portugal. ^27^ Numa fase inicial, os autores analisaram de forma detalhada todos os custos diretos e indiretos relacionados com o procedimento, mostrando que o custo desse procedimento em Portugal é de 22.134,5€ com uma prótese autoexpansível (SEV) e de 23.321,5€ com as próteses expansíveis por balão (BEV). A maior parte do custo relacionou-se com o preço da prótese (SEV 74,5% *versus* BEV 81,5%). Para avaliar o impacto econômico global do procedimento, foram traçados três cenários para a penetração desse tratamento no país. No cenário 1, que considerou a situação em que as taxas de penetração se mantivessem de acordo com as recomendações atuais (189 procedimentos/milhão habitantes), o impacto econômico da TAVI em Portugal seria de 43.770.586€. No cenário 2, em que a indicação para TAVI seria estendida também para os pacientes de risco intermédio, estimou-se que a penetração seria de 241 procedimentos/milhão, o que representaria um impacto econômico de 55.904.116€. Finalmente, no cenário 3, que considera a expansão da utilização desse procedimento para pacientes de baixo risco e idade > 75 anos (penetração de 391 procedimentos/milhão), o impacto seria de 90.754.310€. Em resumo, esse estudo demonstra que a implementação de TAVI para tratamento da estenose aórtica está associada a um impacto econômico muito significativo no SNS, sendo necessário discutir formas de melhorar o acesso ao procedimento, mas mantendo a sustentabilidade do SNS.

## Insuficiência cardíaca e cardiomiopatia

No ano de 2020, a RPC publicou o estudo de Gouveia et al., ^28^ mostrando o impacto econômico da IC. Esse é um tema muito relevante porque a IC é sabidamente a principal causa de custos hospitalares nos Estados Unidos, sendo importante conhecer o seu impacto em cada país. Naquele estudo foram calculados os custos anuais da IC em Portugal incluindo os custos diretos (consumos de recursos) e indiretos (impacto na produtividade da população), tendo por base dados da prática clínica real. Nesse estudo, estimou-se que em 2014 os custos diretos com a IC totalizaram €299 milhões (39% dos custos por internamentos, 24% por medicamentos, 17% por meios complementares de diagnóstico e terapêutica, 16% por consultas e o restante por outras rubricas como urgências e cuidados continuados) e os custos indiretos totalizaram €106 milhões (16% por absenteísmo e 84% por redução de emprego). Esse valor representa 2,6% do total das despesas públicas em saúde. Essa análise incluiu ainda uma projeção dos custos totais da doença até 2036, estimando que esses aumentem significativamente de €405 milhões de euros para €503 milhões, mostrando o importante impacto econômico atual e futuro da IC em Portugal.

Relativamente aos preditores de prognóstico na IC com fração de ejeção reduzida, Ozenc et al., ^29^ publicaram na RPC um artigo interessante que pretendeu avaliar o valor prognóstico do índice de trabalho sistólico ventricular direito (ITSVD). Nesse estudo, foram incluídos prospectivamente 132 pacientes submetidos a cateterismo direito para cálculo do ITSVD. Os autores concluíram que o ITSVD prevê o risco de descompensação cardíaca e que esse parâmetro se relaciona com a classe funcional NYHA em estádios avançados de IC. Esses dados reforçam uma vez mais a importância da avaliação do ventrículo direito nesses pacientes ^30^ e sugerem que é importante a combinação da informação sobre a hemodinâmica do coração direito com a avaliação funcional do ventrículo direito.

Em 2020, na RPC merece ainda destaque o artigo de Menezes et al., ^31^ sobre um tema menos frequente, a técnica de biópsia endomiocárdica. Alguns estudos têm sugerido que a biópsia endomiocárdica ventricular esquerda é mais segura e de superior rentabilidade diagnóstica do que a do ventrículo direito. Nesse estudo, os autores pretenderam avaliar a eficácia, a segurança e a utilidade da realização de biópsia endomiocárdica do ventrículo esquerdo por via transradial num grupo de 27 pacientes. Os autores reportam uma taxa de sucesso de 100% e a ausência de complicações significativas, mostrando a segurança e a boa rentabilidade diagnóstica dessa técnica quando utilizada em pacientes selecionados.

Na IC, existe um cenário de dano oxidativo sistêmico, mas não se sabe como a IC pode afetar diferentes estruturas além do sistema CV, principalmente no dano ao DNA. Desse modo, com o objetivo de estudar o dano ao DNA em diferentes tecidos, como o ventrículo esquerdo, os pulmões e os músculos esqueléticos (diafragma, gastrocnêmio e sóleo), Stefani et al., ^32^ submeteram ratos Wistar machos a ligadura da artéria coronária esquerda com consequente infarto do miocárdio. Os autores observaram que o grupo IC apresentou maior dano ao DNA (% de DNA da cauda, momento da cauda e momento da cauda de Olive) em comparação ao placebo, e o tecido com maior dano foi o sóleo, comparado ao ventrículo esquerdo e ao gastrocnêmio no grupo IC. Concluíram que a IC afeta todos os tecidos, de maneira central e periférica, positivamente correlacionada com a disfunção do ventrículo esquerdo.

A cardiomiopatia crônica da doença de Chagas é frequente no Brasil, causando grave problema de saúde pública. Acredita-se que resulte de miocardite infecciosa de baixo grau, incessante, difusa, com necrose miocitolítica focal e intensa fibrose reativa e reparadora. ^33^ Estima-se que 20-40% dos pacientes com cardiopatia chagásica tenham angina atípica decorrentes de anormalidades na perfusão miocárdica provocadas pelo exercício e reversíveis com o repouso, provavelmente associadas com isquemia microvascular. Campos et al,. ^34^ compararam pacientes com isquemia microvascular relacionada à doença de Chagas com pacientes com isquemia microvascular decorrentes de outras etiologias. Concluíram que os dois grupos tiveram características clínicas, hemodinâmicas e de perfusão miocárdica semelhantes, mas a disfunção global e segmentar do ventrículo esquerdo foi mais grave nos pacientes com isquemia microvascular relacionada com a doença de Chagas.

## Cardiopatias congênitas

Dentre os óbitos por malformações congênitas, os decorrentes de malformação do aparelho circulatório (MAC) apresentam maior impacto sobre a possibilidade de redução da mortalidade por serem evitáveis (com o correto diagnóstico e tratamento) e frequentes. Sua importância relativa aumentou, constituindo-se a terceira causa de óbito em 2015, representando 40% do total. Salim et al., ^35^ avaliaram a distribuição da mortalidade por MAC, por sexo, grupos etários e macrorregiões do Brasil no período de 2000 a 2015, nos menores de 20 anos de idade. Em ambos os sexos, a mortalidade anual por MAC foi de 5,3/100 mil habitantes e a mortalidade proporcional foi de 4,2%. Os autores concluíram que a frequência de diagnósticos imprecisos de óbitos por MAC ainda é elevada em todas as idades, sexos e principalmente nas regiões Norte e Nordeste, constituindo-se em um grave problema de saúde pública no Brasil, pela falta de diagnóstico e tratamento cirúrgico adequado.

Em uma análise das 105.599 cirurgias CV realizadas no Instituto do Coração do Hospital das Clínicas da Faculdade de Medicina da Universidade de São Paulo (InCor), entre janeiro de 1984 e junho de 2019, foi reportada mortalidade global de 5,63%. Em relação às cardiopatias congênitas, houve uma significativa melhora na mortalidade com a implementação do Programa de Melhoria Contínua da Qualidade (PMCQ) e, em 2019, a mortalidade por essa causa foi de 7%. Observou-se também melhora nas cirurgias de revascularização do miocárdio e nas cirurgias valvares com o PMCQ. Cabe ressaltar que as taxas de mortalidade se aproximaram dos padrões internacionais, corroborando a heterogeneidade das mortes por MAC nas diversas regiões do Brasil. ^36^

## Embolia pulmonar

A embolia pulmonar tem apresentação clínica heterogênea e a angio-TC é considerada o método padrão-ouro para o diagnóstico, sendo a dilatação do ventrículo direito o parâmetro mais frequentemente empregado para a estratificação do prognóstico. Esse achado deve ser associado com a dosagem de troponina e da porção N-terminal do peptídeo natriurético do tipo B. ^37^ Soriano et al., ^38^ propuseram que volume vascular pulmonar (VVP) quantificado por *software* automatizado pudesse ser um preditor de mortalidade acurado e de fácil obtenção. Realizaram um estudo de coorte retrospectivo com reanálise da angio-TC de 61 pacientes com embolia pulmonar e calcularam o VVP automaticamente pelo *software* Yacta. Concluíram que o VVP ajustado estimado pelo *software* Yacta parece ser uma ferramenta promissora para a estratificação prognóstica na embolia pulmonar aguda, especialmente se comparado com outros parâmetros prognósticos clássicos da angio-TC.

## Covid-19 e doença cardiovascular

O ano 2020 marcará para sempre a prática da Medicina devido ao enorme impacto da pandemia por COVID-19. As consequências da pandemia nas DCV foram enormes e deixarão marcas durante muitos anos. Por exemplo, num estudo unicêntrico publicado na RPC, observou-se uma redução de 49,7% nas admissões por SCA. ^39^

Também a pandemia reforçou o conceito de que o acometimento cardíaco de pacientes com COVID-19 não é incomum e abrange uma grande variedade de apresentações como arritmias, cardiomiopatias e injúria miocárdica (IM), que se associaram com piores desfechos clínicos. Dois estudos originais unicêntricos demonstraram alta incidência de IM na COVID-19 com impacto em maior mortalidade intra-hospitalar. Nascimento et al., ^40^ mostraram que a IM esteve presente em 36% dos casos de pacientes com COVID-19 internados em terapia intensiva. A hipertensão arterial sistêmica e o IMC foram preditores independentes de risco para a IM e a troponina I US >48,3 ng/ml foi associada com maior mortalidade intra-hospitalar. Almeida Júnior et al., ^41^ demonstraram que, nas primeiras 24 horas de admissão, a troponina T foi marcador independente de mortalidade ou necessidade de ventilação mecânica invasiva em pacientes hospitalizados por COVID-19. Nesse estudo também a proteína C reativa titulada esteve associada independentemente a um pior prognóstico. Os dois estudos ressaltam a importância da IM, demonstrada pela elevação das troponinas I e T, como preditor de mortalidade e efeitos adversos nos pacientes hospitalizados por COVID-19.

Contudo, são ainda mais preocupantes as sequelas que esta crise deixará na sociedade. Num notável artigo publicado na RPC, o General Ramalho Eanes, antigo Presidente da República de Portugal, reflete sobre os impactos na sociedade desta crise sanitária, econômica, social, política e, também, ecológica, nacional e planetária, apelando à necessidade de uma refundação da sociedade como um todo, que deverá no futuro ser “virtuosamente defensável se o eu estiver no nós e o nós no eu, um nós que sejam os outros, todos os outros, homens e outros seres da natureza”. ^42^

## Perspectivas científicas e editoriais

Mais uma vez este esforço conjunto das revistas ABC Cardiol e RPC traz uma provocação apetitosa para o leitor ávido por informação científica atual e original. De grande relevância são os dados específicos da nossa população do Brasil e de Portugal, em especial nos aspectos epidemiológicos da doença arterial coronária e os custos associados a novos procedimentos, como a TAVI.

Outras áreas também tiveram destaque nas edições do ano de 2020, como doenças congênitas, valvares, cardiomiopatias, IC, embolia pulmonar e COVID-19.

Esperamos que este nosso menu selecionado de 2020 desperte no leitor a vontade irresistível de ‘folhear’ digitalmente todas as edições de 2020 dos ABC Cardiol e da RPC ou procurar seu assunto de preferência nas 330 e 138 publicações de 2020, respectivamente.

Finalmente, gostaríamos de reafirmar a relevância desta cooperação científica e editorial quanto às mais importantes publicações de Cardiologia em língua portuguesa.

Abraços a todos e até o ano que vem com os melhores de 2021!

## * Material suplementar

Para informação adicional, por favor, clique aqui.
