# Supplementary material for: O Melhor do Ano 2020 nos Arquivos Brasileiros de Cardiologia e na Revista Portuguesa de Cardiologia
Source: Arq Bras Cardiol. 2021 Jun 8;116(6):1153–60. [Article in Portuguese] doi: 10.36660/abc.20210372 (PMC8288525; doi:10.36660/abc.20210372)
Supplement: Supplementary file 1 [file 2021-0372-material-suplementar.pdf]

Tabela 1 – Lista com a seleção dos dez melhores artigos publicados na *Revista Portuguesa de Cardiologia* em 2020

Autores	Título do artigo
D Abreu et al.[3]	Impact of public health initiatives on acute coronary syndrome fatality rates in Portugal<br>https://www.revportcardiol.org/pt-impact-public-health-initiatives-on-articulo-S0870255119304974
H Dores et al.[6]	Coronary atherosclerotic burden in veteran male recreational athletes with low to intermediate cardiovascular risk<br>https://www.revportcardiol.org/en-coronary-atherosclerotic-burden-in-veteran-articulo-S0870255120303760
D Roque et al.[15]	Understanding a woman's heart: Lessons from 14 177 women with acute coronary syndrome<br>https://www.revportcardiol.org/pt-understanding-woman39s-heart-lessons-from-articulo-S0870255120300858
JP Moura Guedes et al.[19]	P2Y12 inhibitor loading dose before catheterization in ST-segment elevation myocardial infarction: Is this the best strategy?<br>https://www.revportcardiol.org/en-p2y12-inhibitor-loading-dose-before-articulo-S2174204920302026
J Santos-Faria et al.[24]	MicroRNAs and ventricular remodeling in aortic stenosis<br>https://www.revportcardiol.org/pt-micrornas-ventricular-remodeling-in-aortic-articulo-S0870255120302675
C Guerreiro et al.[26]	Short and long-term clinical impact of transcatheter aortic valve implantation in Portugal according to different access routes: Data from the Portuguese National Registry of TAVI<br>https://www.revportcardiol.org/en-short-long-term-clinical-impact-transcatheter-articulo-S217420492030413X
R Fontes-Carvalho et al.[27]	Present and future economic impact of transcatheter aortic valve replacement on the Portuguese national healthcare system<br>https://www.revportcardiol.org/pt-present-future-economic-impact-transcatheter-articulo-S0870255120303358
M Gouveia et al.[28]	Current costs of heart failure in Portugal and expected increases due to population aging<br>https://www.revportcardiol.org/en-current-costs-heart-failure-in-articulo-S217420492030074X
E Ozenc et al.[29]	Impact of right ventricular stroke work index on predicting hospital readmission and functional status of patients with advanced heart failure<br>https://www.revportcardiol.org/en-impact-right-ventricular-stroke-work-articulo-S2174204920303603
M Nobre Menezes et al.[31]	Transradial left ventricular endomyocardial biopsy feasibility, safety and clinical usefulness: Initial experience of a tertiary university center<br>https://www.sciencedirect.com/science/article/pii/S0870255120302845
