# Supplementary material for: O Melhor do Ano 2020 nos Arquivos Brasileiros de Cardiologia e na Revista Portuguesa de Cardiologia
Source: Arq Bras Cardiol. 2021 Jun 8;116(6):1153–60. [Article in Portuguese] doi: 10.36660/abc.20210372 (PMC8288525; doi:10.36660/abc.20210372)
Supplement: Translation

# Translation: 2020 Top 10 Original Articles in the *Arquivos Brasileiros de Cardiologia* and the *Revista Portuguesa de Cardiologia*

**Authors:** Ricardo Fontes-Carvalho, Gláucia Maria Moraes de Oliveira, Nuno Cardim, Carlos Eduardo Rochitte

**Affiliations:** 1 Portugal Departamento de Cardiologia – Centro Hospitalar de Vila Nova de Gaia/Espinho, Vila Nova de Gaia - Portugal; 2 Portugal Departamento de Cirurgia e Fisiologia – Faculdade de Medicina – Universidade do Porto, Porto - Portugal; 3 Brasil Faculdade de Medicina – Universidade Federal do Rio de Janeiro, Rio de Janeiro, RJ - Brasil; 4 Brasil Instituto do Coração Edson Saad – Universidade Federal do Rio de Janeiro, Rio de Janeiro, RJ - Brasil; 5 Brasil Instituto do Coração (InCor) – Faculdade de Medicina da Universidade de São Paulo, São Paulo, SP - Brasil; 6 Brasil Hospital do Coração (HCOR), São Paulo, SP - Brasil; 7 Portugal Hospital da Luz-Lisboa - Faculdade de Ciências Médicas da Universidade Nova de Lisboa, Lisboa - Portugal

**Keywords:** Cardiovascular Diseases, Technical Cooperation/trends, Researcher Exchange/trends, Information Dissemination, Journal of Impact Factor, Periodicals as Topic, Portugal, Brazil

## Introduction

In recent years, the *Revista Portuguesa de Cardiologia* (RPC) and the *Arquivos Brasileiros de Cardiologia* (ABC Cardiol) have gotten together annually to elaborate a list of the most relevant papers published in both journals,^1 , 2^ highlighting some of the best scientific articles in Portuguese.

Based on the success of that initiative, the editors of both journals have decided to cooperate once again to elaborate the ‘Top 10’ list of 2020 of each journal. The year 2020 was marked by the huge impact of the COVID-19 pandemic in both countries, with an enormous pressure on the healthcare institutions and professionals. Despite those challenges, the scientific quality of the publications in both journals remained extremely high, with excellent original articles.

The selection of the best publications is always complex and sometimes can be imperfect and even unfair, and the process occurred independently of the citations obtained by the articles. This selection included only original articles; no review was considered.

We briefly present the major results of the articles selected along with their scientific relevance. Tables 1 and 2 list the most important papers published in 2020.

**Table 1:** – List of the ten best articles published in the *Revista Portuguesa de Cardiologia* in 2020

Authors	Title of the article
D Abreu et al.^3^	Impact of public health initiatives on acute coronary syndrome fatality rates in Portugal
H Dores et al.^6^	Coronary atherosclerotic burden in veteran male recreational athletes with low to intermediate cardiovascular risk
D Roque et al.^15^	Understanding a woman's heart: Lessons from 14 177 women with acute coronary syndrome
JP Moura Guedes et al.^19^	P2Y12 inhibitor loading dose before catheterization in ST-segment elevation myocardial infarction: Is this the best strategy?
J Santos-Faria et al.^24^	MicroRNAs and ventricular remodeling in aortic stenosis
C Guerreiro et al.^26^	Short and long-term clinical impact of transcatheter aortic valve implantation in Portugal according to different access routes: Data from the Portuguese National Registry of TAVI
R Fontes-Carvalho et al.^27^	Present and future economic impact of transcatheter aortic valve replacement on the Portuguese national healthcare system
M Gouveia et al.^28^	Current costs of heart failure in Portugal and expected increases due to population aging
E Ozenc et al.^29^	Impact of right ventricular stroke work index on predicting hospital readmission and functional status of patients with advanced heart failure
M Nobre Menezes et al.^31^	Transradial left ventricular endomyocardial biopsy feasibility, safety and clinical usefulness: Initial experience of a tertiary university center

**Table 2:** – List of the ten best articles published in the *Arquivos Brasileiros de Cardiologia* em 2020

Authors	Title of the article
Soriano L^38^	1. Volume Vascular Pulmonar Estimado por Software Automatizado é um Preditor de Mortalidade após Embolia Pulmonar Aguda Pulmonary Vascular Volume Estimated by Automated Software is a Mortality Predictor after Acute Pulmonary Embolism http://abccardiol.org/wp-content/uploads/articles_xml/1678-4170-abc-115-05-0809/1678-4170-abc-115-05-0809.x64000.pdf http://abccardiol.org/wp-content/uploads/articles_xml/1678-4170-abc-115-05-0809/1678-4170-abc-115-05-0809-en.x64000.pdf
Oliveira-Junior SA^11^	2. Bloqueio de Receptores AT_1_Melhora o Desempenho Funcional Miocárdico na Obesidade AT_1_Receptor Blockade Improves Myocardial Functional Performance in Obesity http://abccardiol.org/wp-content/uploads/articles_xml/0066-782X-abc-115-01-0017/0066-782X-abc-115-01-0017.x64000.pdf http://abccardiol.org/wp-content/uploads/articles_xml/0066-782X-abc-115-01-0017/0066-782X-abc-115-01-0017-en.x64000.pdf
Salim TR^35^	3. Desigualdades nas Taxas de Mortalidade por Malformações do Sistema Circulatório em Crianças Menores de 20 Anos de Idade entre Macrorregiões Brasileiras Inequalities in Mortality Rates from Malformations of Circulatory System Between Brazilian Macroregions in Individuals Younger Than 20 Years http://abccardiol.org/wp-content/uploads/articles_xml/0066-782X-abc-115-06-1164/0066-782X-abc-115-06-1164.x64000.pdf http://abccardiol.org/wp-content/uploads/articles_xml/0066-782X-abc-115-06-1164/0066-782X-abc-115-06-1164-en.x64000.pdf
Mejia OAV^36^	4. Análise de >100.000 Cirurgias Cardiovasculares Realizadas no Instituto do Coração e a Nova Era com Foco nos Resultados Analysis of >100,000 Cardiovascular Surgeries Performed at the Heart Institute and a New Era of Outcomes http://abccardiol.org/wp-content/uploads/articles_xml/0066-782X-abc-S0066-782X2020000400603/0066-782X-abc-S0066-782X2020000400603-pt.x64000.pdf http://abccardiol.org/wp-content/uploads/articles_xml/0066-782X-abc-S0066-782X2020000400603/0066-782X-abc-S0066-782X2020000400603.x64000.pdf
Alves L^22^	5. Hospitalização por Infarto Agudo do Miocárdio: Um Registro de Base Populacional Hospitalization for Acute Myocardial Infarction: A Population-Based Registry http://abccardiol.org/wp-content/uploads/articles_xml/1678-4170-abc-115-05-0916/1678-4170-abc-115-05-0916.x64000.pdf http://abccardiol.org/wp-content/uploads/articles_xml/1678-4170-abc-115-05-0916/1678-4170-abc-115-05-0916-en.x64000.pdf
Campos FA^34^	6. Estudo Comparativo da Doença Coronariana Microvascular Causada por Doença de Chagas com Outras Etiologias Chagas Cardiomyopathy as the Etiology of Suspected Coronary Microvascular Disease. A Comparison Study with Suspected Coronary Microvascular Disease of Other Etiologies http://abccardiol.org/wp-content/uploads/articles_xml/0066-782X-abc-115-06-1094/0066-782X-abc-115-06-1094.x64000.pdf http://abccardiol.org/wp-content/uploads/articles_xml/0066-782X-abc-115-06-1094/0066-782X-abc-115-06-1094-en.x64000.pdf
Stefan GP^32^	7. Quantificação de Dano em DNA em Diferentes Tecidos em Ratos com Insuficiência Cardíaca Quantification of DNA Damage in Different Tissues in Rats with Heart Failure http://abccardiol.org/wp-content/uploads/articles_xml/0066-782X-abc-S0066-782X2020000200234/0066-782X-abc-S0066-782X2020000200234-pt.x64000.pdf http://abccardiol.org/wp-content/uploads/articles_xml/0066-782X-abc-S0066-782X2020000200234/0066-782X-abc-S0066-782X2020000200234.x64000.pdf
Basilio PG^10^	8. Dieta Intermitente Atenua a Remodelação Cardíaca Causada pelo Exercício Físico Intermittent Fasting Attenuates Exercise Training-Induced Cardiac Remodeling http://abccardiol.org/wp-content/uploads/articles_xml/0066-782X-abc-115-02-0184/0066-782X-abc-115-02-0184.x64000.pdf http://abccardiol.org/wp-content/uploads/articles_xml/0066-782X-abc-115-02-0184/0066-782X-abc-115-02-0184-en.x64000.pdf
Santos JL^12^	9. Os Percentis e Pontos de Corte da Circunferência Abdominal para Obesidade em uma Ampla Amostra de Estudantes de 6 a 10 Anos de Idade do Estado de São Paulo, Brasil Waist Circumference Percentiles and Cut-Off Values for Obesity in a Large Sample of Students from 6 To 10 Years Old Of The São Paulo State, Brazil http://abccardiol.org/wp-content/uploads/articles_xml/0066-782X-abc-114-03-0530/0066-782X-abc-114-03-0530.x64000.pdf http://abccardiol.org/wp-content/uploads/articles_xml/0066-782X-abc-114-03-0530/0066-782X-abc-114-03-0530-en.x64000.pdf
Ferreira LCM^21^	10. Mortalidade por Infarto Agudo do Miocárdio no Brasil de 1996 a 2016: 21 Anos de Contrastes nas Regiões Brasileiras Mortality Due to Acute Myocardial Infarction in Brazil from 1996 to 2016: 21 Years of Disparities in Brazilian Regions http://abccardiol.org/wp-content/uploads/articles_xml/1678-4170-abc-115-05-0849/1678-4170-abc-115-05-0849.x64000.pdf http://abccardiol.org/wp-content/uploads/articles_xml/1678-4170-abc-115-05-0849/1678-4170-abc-115-05-0849-en.x64000.pdf

## Prevention and cardiovascular risk

Cardiovascular disease (CVD) is the leading cause of death in Portugal, Brazil, and developed countries. Therefore, the implementation of public health measures directed to the general population is fundamental to reduce the impact of CVD on society. In the years 2000-2010, Portugal implemented a set of public health policies to reduce mortality from CVD. Some examples of such measures were the smoking ban in 2008, the salt reduction regulation in 2010, and the implementation of the fast-track system to the coronary unit in 2007. In a study published in the RPC in 2020, *Abreu* et al.^3^ assessed the impact of those three health policies on the reduction of the case-fatality rate from acute coronary syndrome (ACS), analyzing the epidemiological CVD data from 2000 to 2016. That study has suggested that the smoking ban and the coronary fast-track system implementation had an immediate decrease in the ACS case-fatality rate, which was not observed with the salt reduction regulation. The reduction in salt intake is known to mainly reduce the risk of stroke, which might explain the results of that study. In addition, those data are consistent with those observed in the Portuguese National Registry of Acute Coronary Syndromes, showing a consistent increase in revascularization rates after the coronary fast-track system implementation.^4^ Another study published in the RPC in 2020 showed that a comprehensive smoking ban in the Community of Valencia (Spain) associated with a marked reduction in the adjusted hospitalization rates for myocardial infarction.^5^ This type of study and analysis are fundamental to the implementation of new public health measures to reduce the impact of CVD on society.

Still regarding cardiovascular (CV) risk, the RPC has published an interesting study by Dores et al.^6^ assessing the coronary atherosclerotic burden of 105 asymptomatic veteran male athletes with low to intermediate CV risk. Those athletes were mainly engaged in high-dynamic sports and underwent cardiac computed tomography (CT) for coronary artery calcium scoring and computed tomography angiography (CT angiography). Although that population seemed “healthy”, that study showed a high coronary atherosclerotic burden in 25.7% of the athletes and coronary obstructive lesions in 5.7%. The extent and severity of coronary plaques did not relate to exercise volume or type. Those data are important to understand the best screening strategies in veteran athletes^7^ and raise the possibility of using coronary CT for the routine assessment of those individuals, as discussed in the editorial by Pelliccia about that article.^8^

Regarding the age-standardized mortality rates from CVD attributable to risk factors in 2018 in Brazil, for women and men, dietary risks rank second, behind only arterial hypertension.^9^ Basilio et al.^10^ have studied the influence of the combination of intermittent fasting (with calorie restriction) and physical exercise on physical functioning, glucose metabolism, and cardiac remodeling in male Wistar rats for 12 weeks. Those authors have hypothesized that physical exercise would increase physical performance and attenuate myocardial remodeling because of intermittent calorie restriction. They have observed that physical exercise increased physical functioning and promoted cardiac fibrosis. They have concluded that intermittent fasting associated with improved glucose tolerance and attenuated cardiac remodeling induced by physical exercise but did not interfere in physical functioning.

The increase in body mass index (BMI), representing the changes observed in obesity, is the third risk factor for women and the fourth for men, in the previously mentioned ranking.^9^ Oliveira-Júnior et al.^11^ have hypothesized that obesity would associate with changes in myocardial functional performance sustained in different stimulation conditions and reduced by AT1-receptor blockade. Those authors have studied Wistar rats fed either a control or high-fat diet and divided according to the presence of obesity. The obese and control groups received losartan (30 mg/kg/day) in drinking water for four weeks. Those authors have concluded that diet-induced obesity promoted cardiac remodeling, sustained by ventricular hypertrophy and myocardial dysfunction, confirming the initial hypothesis that the stimulation of AT1 receptors would associate with myocardial dysfunction in obese rats. Losartan improved the myocardial function of rats with diet-induced obesity.

It is worth noting that obesity has increased not only in the adult population, but in children and adolescents worldwide as well. The measure of waist circumference, which is an easily obtainable parameter, has high sensitivity to predict children’s visceral fat levels. Santos et al.^12^ have performed a multicenter, prospective, cross-sectional study with 22 000 children (11 199 boys) aged 6-10 years, registered at public and private elementary schools in 13 cities of São Paulo State. They measured height, weight, and waist circumference. The prevalence of obesity ranged from 17% (6 years of age) to 21.6% (9 years of age) among boys and from 14.1% (7 years of age) to 17.3% (9 years of age) among girls. That study confirms the findings on childhood obesity from the ERICA Study,^13^ highlighting the importance of early intervention in dietary risks and obesity to prevent CV deaths in adulthood.

## Coronary artery disease and acute coronary syndrome

Although mortality from coronary artery disease has decreased in recent years, several studies have reported the persistence of great differences in diagnosis and treatment of coronary artery disease between genders,^14^ suggesting that women often undergo worse treatment. In a study published in the RPC, including 49 113 patients (34 936 men and 14 177 women), Roque et al.^15^ have assessed the differences between genders in the treatment of ACS by using data from the Portuguese National Registry on Acute Coronary Syndromes. Those authors have observed that, as compared to men, women are more frequently admitted for non-ST elevation ACS and more often have atypical symptoms. That might explain the women’s longer time from symptom onset to reperfusion. The risk of in-hospital mortality was significantly higher in the female sex (OR 1.94; 95%CI: 1.78-2.12) as was their risk of major bleeding, heart failure (HF), atrial fibrillation, mechanical complications, and cardiogenic shock. It is worth noting that women were less likely to receive the recommended secondary prevention therapies, both during hospitalization and at hospital discharge. Briefly, as discussed in the Thomas Lüscher’s editorial about that article,^16^ those data show the importance of shedding light on the theme of inequality between genders in the treatment of CVD and highlight the need to implement specific measures that can reduce the difference in treatment between genders.

In another study published in the RPC in 2020 based on data from the Portuguese National Registry of Acute Coronary Syndromes, the authors have assessed one of the major themes in current scientific discussion, the optimal time to administer the loading dose of an antiplatelet agent (P2Y12 inhibitor) to patients with ST-elevation ACS: before or during catheterization.^17 , 18^ For that analysis, 4 123 patients with ACS were included, 66% of whom received the P2Y12 inhibitor before catheterization.^19^ Multivariate analysis showed that patients who received the P2Y12 inhibitor before catheterization had a significant increase in the composite bleeding endpoint (major bleeding, need for transfusion or hemoglobin drop > 2g/dL), hemoglobin drop > 2g/dL, and reinfarction, in addition to no benefit regarding reduction in major CV adverse events (MACE) or in-hospital death. These data are similar to those observed in other registries,^20^ contributing, thus, to this important discussion.

Acute myocardial infarction is also the leading cause of death in Brazil, where regional and gender-related disparities in the temporal trends of mortality rates in most recent years have been observed. Ferreira et al.^21^ have conducted a 21-year time-series study (1996-2016) using data from the Brazilian Mortality Information System (SIM) with corrections for ill-defined causes of death, garbage codes, and underreporting. The authors have observed that mortality decreased more significantly in the female sex (–2.2%; 95% CI: –2.5; –1.9) than in the male sex (–1.7%; 95% CI: –1.9; –1.4) and more in the capitals (–3.8%; 95% CI: –4.3; –3.3) than in the inner areas (–1.5%; 95% CI: –1.8; –1.3). In addition, they have found regional differences with increase for men living in the inner areas of the Northern (3.3; 95% CI: 1.3; 5.4) and Northeastern (1.3%; 95% CI: 1.0; 1.6) regions. They have concluded that the corrections of the number of deaths are essential to more reliable estimates on the myocardial infarction mortality trends in Brazil.

Of the ischemic heart diseases, ST-elevation myocardial infarction (STEMI) has the highest proportional mortality. Population-based studies on hospitalizations from that cause in Brazil are scarce. Alves L & Polanczyk CA^22^ have performed a population-based prospective cohort study with consecutive registries of hospitalizations for STEMI in a Brazilian southern city from 2011 to 2014. They reported an annual incidence of 108 cases/100 000 inhabitants with reperfusion rate of 80.9%, in-hospital mortality of 8.9%, and CV event rate of 6.1%. Those authors have concluded that, compared to developed countries, Brazil had a higher number of hospitalizations, but the therapeutic approach and in-hospital mortality were similar to those of developed countries.

## Valvular diseases

Aortic valve stenosis is currently the most frequent valvular disease in the western world and its prevalence will continue to increase exponentially due to population aging.^23^ Aortic stenosis changes the left ventricular structure and function, and several mechanisms are involved in its pathophysiology. In a study published in 2020 in the RPC, Santos-Faria et al.^24^ have assessed the role of microRNA post-transcriptional modulation in the appearance of hypertrophy and myocardial fibrosis. Analyzing the myocardial biopsies of 11 patients undergoing aortic valve replacement, those authors have observed that microRNA-101-3p and microRNA-4268 have potential new roles in the hypertrophic response of patients with aortic stenosis and may be used as predictors of post-surgery reverse remodeling. In addition, the role of those microRNAs in the regulation of the renin-angiotensin-aldosterone system may help find new therapeutic targets for hypertrophy regression.^25^

Transcatheter aortic valve implantation (TAVI) has changed the paradigm of severe aortic stenosis treatment. In 2020, the RPC published an article assessing the short- and long-term results of TAVI in Portugal, using data from the Portuguese National Registry of TAVI, in an analysis of 2346 procedures.^26^ In general, TAVI associated with high efficacy and safety, with a 30-day mortality rate of 4.8%. The predictors of 30-day mortality were peripheral arterial disease, previous angioplasty, left ventricular dysfunction, and NYHA functional class III-IV. The predictors of one-year mortality were NYHA functional class III-IV, non-transfemoral route and life-threatening bleeding. In addition, the type of route (transfemoral or another) was analyzed, showing the association of transapical approach with higher mortality and higher risk of complications, related to the patient’s profile (severity and more comorbidities).

Despite its benefits, the penetration rate of TAVI in Portugal is still low, with rates much lower than the means of the European Union. In addition, TAVI has high costs, being important to ensure the sustainability of the Portuguese healthcare system. Another article on the same theme published in the RPC in 2020 analyzed the current and future economic impact of TAVI in Portugal.^27^ In the initial phase, the authors analyzed all direct and indirect costs related to TAVI, showing that its costs in Portugal were 22 134.5€ for the self-expanding valves (SEV) and 23 321.5€ for the balloon-expanding valves (BEV). Most of the cost related to the price of the prosthesis (SEV 74.5% *versus* BEV 81.5%). To assess the global economic impact of the procedure, three scenarios were constructed. In scenario 1, with TAVI penetration rates according to current guidelines (189 procedures/million inhabitants), the economic impact of TAVI in Portugal would be 43 770 586€. In scenario 2, with TAVI indication extending to intermediate-risk patients (estimated penetration of 241 procedures/million inhabitants), the economic impact would be 55 904 116€. In scenario 3, with TAVI indication extending to low-risk patients aged > 75 years (penetration of 391 procedures/million inhabitants), the economic impact would be 90 754 310€. Briefly, that study shows that the implementation of TAVI to treat aortic stenosis is associated with a significant economic impact on the Portuguese healthcare system; thus, ways to improve access to the procedure should be discussed but maintaining the sustainability of the healthcare system.

## Heart failure and cardiomyopathy

In 2020, the RPC published a study by Gouveia et al.^28^ on the economic impact of HF, because, knowing that HF is the major responsible for hospital costs in the United States, its impact should be known in every country. Those authors calculated the annual costs of HF in Portugal, including direct (resource consumption) and indirect (productivity losses) costs, based on data from real clinical practice. In that study, the direct costs with HF in 2014 were €299 million (39% for hospitalizations, 24% for medicines, 17% for diagnostic and therapeutic tests, 16% for consultations, and the rest for other needs, such as emergencies and long-term care). The indirect costs were €106 million (16% for absenteeism and 84% for reduced employment). Those values represent 2.6% of total public health expenditure. In addition, the projection of total costs of HF up to 2036, estimating they significantly increase from €405 million to €503 million, shows the importance of current and future economic impact of HF in Portugal.

Regarding the prognostic predictors of HF with reduced ejection fraction, Ozenc et al.^29^ have published in the RPC an article assessing the prognostic value of the right ventricular stroke work index (RVSWI). That study prospectively enrolled 132 patients undergoing right heart catheterization for RVSWI calculation. Those authors concluded that RVSWI predicts the risk of cardiac decompensation and correlates with NYHA functional class in advanced stages of HF. Those data reinforce the relevance of right ventricular assessment in those patients^30^ and suggest the importance of combining the information on right cardiac hemodynamics with that of right ventricular function.

It is worth noting the article by Menezes et al.^31^ published in 2020 in the RPC about a theme that has not received much attention in the literature, the endomyocardial biopsy. Some studies have suggested that left ventricular endomyocardial biopsy is safer and has a higher diagnostic yield than that of the right ventricle. Those authors have assessed the efficacy, safety, and usefulness of performing transradial left ventricular endomyocardial biopsy in a group of 27 patients. Those authors have reported a success rate of 100% and no significant complications, showing the safety and good diagnostic yield of the technique when used in properly selected patients.

There is a background of systemic oxidative damage in HF, but how HF can affect different structures other than the CV system, mainly DNA damage, is yet to be known. Thus, aiming at assessing DNA damage in different tissues, such as the left ventricle, lungs, and skeletal muscles (diaphragm, gastrocnemius, and soleus), Stefani et al.^32^ have submitted male Wistar rats to left coronary artery ligation with consequent myocardial infarction. Those authors have reported higher DNA damage (% tail DNA, tail moment, and Olive tail moment) in the HF group as compared to the placebo group, and soleum was the most damaged tissue as compared to left ventricle and gastrocnemius in the HF group. They have concluded that HF affects all tissues, centrally and peripherally, in addition to being positively correlated with left ventricular dysfunction.

Chronic cardiomyopathy from Chagas disease is frequent in Brazil, causing severe public health problems. It is believed to result from persistent, diffuse, low-grade infectious myocarditis with focal myocytolysis and intense reactive and reparative fibrosis.^33^ It is estimated that 20-40% of patients with Chagas heart disease have atypical angina resulting from myocardial perfusion abnormalities caused by exercise that reverse at rest, probably associated with microvascular ischemia. Campos et al.^34^ have compared patients with Chagas disease-related microvascular ischemia to patients with microvascular ischemia of other etiologies. They have concluded that the clinical, hemodynamic, and myocardial perfusion characteristics were similar in both groups but left ventricular segmental and global dysfunction was more severe in patients with Chagas disease-related microvascular ischemia.

## Congenital heart diseases

Of the deaths from congenital malformations, those from malformations of the circulatory system (MCS) have a higher impact on the possibility of mortality reduction because they are preventable (if correctly diagnosed and treated) and frequent. Their relative importance has increased, and they became the third cause of death in 2015, representing 40% of the total. Salim et al.^35^ have assessed the distribution of mortality from MCS, according to sex, age groups, and Brazilian geographical regions, from 2000 to 2015, in individuals under the age of 20 years. In both sexes, the annual mortality from MCS was 5.3/100 000 inhabitants and proportional mortality was 4.2%. Those authors have concluded that the frequency of misdiagnosis of deaths from MCS is high at all ages, both sexes, and mainly in the Northern and Northeastern regions. That is a severe public health problem in Brazil, because of the lack of diagnosis and proper surgical treatment.

In an analysis of 105 599 CV surgeries performed at the Heart Institute of the Hospital das Clínicas of the University of São Paulo (InCor), between January 1984 and June 2019, a 5.63% global mortality has been reported. Regarding congenital heart diseases, a significant improvement in mortality has been observed after the implementation of the Continuous Quality Improvement Program (CQIP), and, in 2019, mortality from that cause was 7%. In addition, improvement was observed in coronary artery bypass grafting and valvular surgeries with the CQIP. It is worth noting that the mortality rates have come closer to international standards, corroborating the heterogeneity of the deaths from MCS in different Brazilian regions.^36^

## Pulmonary embolism

Pulmonary embolism has a heterogeneous clinical presentation, and CT angiography is the gold-standard method for its diagnosis, with right ventricular dilation being the most frequently used parameter for prognostic stratification. That finding should be associated with the measurement of troponin and N-terminal B-type natriuretic peptide.^37^ Soriano et al.^38^ have proposed that pulmonary vascular volume (PVV), automatically estimated using a software, could be an accurate and easily obtainable mortality predictor. Those authors have conducted a retrospective cohort study with reanalysis of the CT angiography of 61 patients with pulmonary embolism and calculation of the PVV automatically using the Yacta software. They have concluded that adjusted PVV estimated by using that software seems a promising tool for prognostic stratification in acute pulmonary embolism, especially when compared to other classic prognostic parameters of CT angiography.

## Covid-19 and cardiovascular disease

The year 2020 has changed forever the medical practice because of the huge impact of the COVID-19 pandemic, whose repercussions on CVD have been enormous and will be felt for years. A single-center study published in the RPC has reported a 49.7% reduction in ACS admissions.^39^

In addition, the pandemic has reinforced the concept that the cardiac impairment of patients with COVID-19 is not uncommon and comprises a wide range of presentations, such as arrhythmias, cardiomyopathies, and myocardial injury (MI), all associated with worse clinical outcomes. Two original single-center studies have shown a high incidence of MI in COVID-19, associated with higher in-hospital mortality. Nascimento et al.^40^ have shown the presence of MI in 36% of the patients with COVID-19 in the intensive care unit. Systemic arterial hypertension and BMI were independent predictors of risk for MI, and high-sensitivity troponin I >48.3 ng/mL was associated with higher in-hospital mortality. Almeida Júnior et al.^41^ have shown that, within the first 24 hours from admission, troponin T was an independent marker of mortality or need for invasive mechanical ventilation in patients admitted with COVID-19. In addition, titrated C reactive protein was independently associated with worse prognosis. Both studies have emphasized the importance of MI, evidenced by the elevation in troponins I and T, as a predictor of mortality and adverse effects in patients admitted with COVID-19.

However, the sequelae from this crisis are even more frightening. In a remarkable article published in the RPC, General Ramalho Eanes, former president of Portugal, reflects on the impacts on society of this sanitary, economic, social, political, ecological, national, and planetary crisis. He emphasizes the need for rethinking society as a whole to integrate the individual and the group, encompassing all living things.^42^

## Scientific and editorial perspectives

Once again, this joint effort of the journals ABC Cardiol and RPC presents a teaser for the reader avid for updated and original scientific information. The specific data on the population from Brazil and Portugal are highly relevant, especially regarding the epidemiological aspects of coronary artery disease and the costs associated with new procedures, such as TAVI.

Other areas have been highlighted in the 2020 issues, such as congenital and valvular diseases, cardiomyopathies, HF, pulmonary embolism, and COVID-19.

We hope this selected 2020 menu triggers the readers’ irresistible desire to digitally browse all 2020 ABC Cardiol and RPC issues, searching for their preferred subjects out of the 330 and 138 publications in 2020, respectively.

Finally, we reassure the relevance of this scientific and editorial cooperation regarding the most important publications in cardiology in Portuguese.

Best regards to you all, looking forward to select the 2021 Top 10!

## * Supplemental Materials

For additional information, please click here.
